# Guillain-Barré Syndrome following Treatment with Sunitinib Malate

**DOI:** 10.1155/2014/712040

**Published:** 2014-06-11

**Authors:** Ziad Kanaan, Zain Kulairi, Mirela Titianu, Sandip Saha, Sarwan Kumar

**Affiliations:** Department of Internal Medicine, Crittenton Hospital Medical Center, Wayne State University, Rochester, MI 48307, USA

## Abstract

Sunitinib malate (Sutent, SU011248) is an oral multitargeted tyrosine kinase inhibitor (TKI) used for the treatment of metastatic renal cell carcinoma and imatinib (Gleevec)—resistant gastrointestinal stromal tumor (GIST) with few reported side effects including asthenia, myelosuppression, diarrhea, and mucositis. Scarce literature exists regarding the rare but often serious toxicities of sunitinib. Autoimmune and neurological side effects have been linked to sunitinib's inhibition of VEGF receptors with a corresponding increase in VEGF levels, which is associated with development of different neuropathies. We hereby report an interesting case of Guillain-Barré syndrome in a middle-aged patient with metastatic renal cell carcinoma following sunitinib treatment.

## 1. Introduction

Sunitinib malate (Sutent, SU011248) is an oral multitargeted tyrosine kinase inhibitor (TKI) and usually used for the treatment of metastatic renal cell carcinoma and imatinib (Gleevec)—resistant gastrointestinal stromal tumor (GIST) [1, 2]. The most common reported side effects of sunitinib usually include asthenia, myelosuppression, diarrhea, mucositis, nausea, altered taste, and skin changes [3]. Scarce literature exists regarding the rare but often serious toxicities of sunitinib. We hereby report an interesting case of sunitinib-induced Guillain-Barré syndrome (GBS) in a patient with metastatic renal cell carcinoma following treatment with sunitinib.

## 2. Case

We report the case of a middle-aged patient known to have a significant history of left renal cell carcinoma diagnosed 2 decades ago with subsequent nephrectomy. Patient was known to have multiple comorbidities including type-II diabetes mellitus, hypertension, chronic kidney disease, hyperlipidemia, gout, and osteoarthritis. Prior to presentation, the patient reported to have experienced intractable nausea that persisted for 7 days, along with feeling bloated and early satiety. Patient reported a 12-pound weight loss during this period. On presentation, patient was afebrile and hemodynamically stable and an abdominal ultrasound was performed which showed extensive masses of different sizes throughout the liver, consistent with extensive metastatic disease. The largest mass measured approximately 4.3 cm in diameter and the liver was diffusely enlarged with no splenomegaly. Eventually, the patient underwent a CT-guided percutaneous needle biopsy and the histocytology report was consistent with metastatic carcinoma, with renal carcinoma being the primary. The patient was discharged 2 days later and followed up with an oncologist who started him on sunitinib for treatment of his metastatic disease. Following treatment with sunitinib one month later, the patient began to experience weakness in both of his legs and reported falling two times without loss of consciousness. Over a period of 1 week, the patient became bedridden due to decreased motor power and sensation of his lower extremities. On the other hand, no upper extremity weakness or decreased sensation was reported. MRI of the upper and lower back was not possible because he was claustrophobic. Blood levels including those of acetylcholine receptor and voltage-gated calcium channel antibodies were normal, thus ruling out myasthenia gravis and Lambert-Eaton syndrome. A lumbar puncture revealed abdominal albumin-cytologic dissociation with a white blood cell count of 1 and a protein level of 177. Clinical picture was consistent with the diagnosis of GBS and the patient was treated with plasmapheresis for 7 days and reported significant improvement. Eventually, the patient underwent physical rehabilitation and was discharged in good condition.

## 3. Discussion

Guillain-Barré syndrome (GBS) is one of the most common reasons of acute polyneuropathy in adults. Pathogenesis of GBS is unknown, but it is generally accepted that it results from an aberrant humor and cellular immune response against components of the peripheral nervous system [4]. The development of GBS in our patient could be linked to treatment with sunitinib as reported in the medical literature [3]. The Naranjo probability score [5] for determining the likelihood of whether an adverse drug reaction is actually due to the drug (sunitinib in this case) rather than the result of other factors was calculated. The score was +6 suggesting probable adverse reaction due to sunitinib [5].

Sunitinib is usually metabolized by cytochrome P450 (CYP) 3A4 to an active metabolite, SU12662, which is further metabolized by CYP3A4 to an inactive moiety [1]. The parent compound and active metabolite have similar biochemical activity and potency and reach similar plasma concentrations [1]. Sunitinib and SU12662 have a half-life of 40 to 60 hours and 80 to 110 hours, respectively [1]. Steady-state concentrations of both active entities are reached after 10 to 14 days of therapy [1].

At the molecular level, sunitinib inhibits members of the split-kinase domain family of receptor tyrosine kinases (RTKs) including the vascular endothelial growth factor receptors (VEGFRs) types 1 and 2 (FLT1 and FLK1 or KDR); platelet-derived growth factor receptors alpha and beta (PDGFR-alpha and PDGFR-beta); the stem cell factor receptor c-KIT; and the* FLT3* and* RET* kinases [1, 6]. Clinically, the inhibition of these RTKs results in a reduction of crucial tumor-specific characteristics like growth, progression, metastases, and angiogenesis [7].

Autoimmune and neurological side effects have been linked to sunitinib's inhibition of VEGF receptors with a corresponding increase in VEGF levels [8]. Sunitinib may damage the capillary endothelium by targeting VEGFR and PDGFR [8]. A possible explanation is that VEGF expression in the microvasculature adjacent to the choroid plexus epithelium is pivotal for the maintenance of the choroid plexus structure [8]. Consequently, the inhibition of the VEGF signaling pathway may result in loss of the choroid plexus structure [8]. The impact of VEGF inhibitors on the immune system has been elucidated in other animal model studies, where VEGF inhibited the development of dendritic cells and increased B-lymphocytes and immature myeloid cells [8].

Further evidence about sunitinib's neurological side effects is elucidated through a study examining the levels of the vascular endothelial growth factor/vascular permeability factor (VEGF) in the serum and cerebrospinal fluid (CSF) from 10 patients with PEOMS syndrome (syndrome of polyneuropathy, organomegaly, endocrinopathy, M-protein, and skin changes) [9]. Serum VEGF levels were found to be 15–30 times those in control subjects or patients with GBS, chronic inflammatory demyelinating polyneuropathy (CIDP), and other neurological disorders [9]. The CSF VEGF levels, however, were similar to those found in GBS and CIDP [9]. The results of this study suggest that the overproduction of VEGF is important in the pathogenesis of this disorder [9, 10].

## 4. Conclusion

Sunitinib has an inhibitory extracellular action against VEGF receptors, thus increasing VEGF levels, which is commonly associated with development of different neuropathies. In the majority of cases, the benefits of sunitinib far outweigh the rare emerging toxicities; however, physicians prescribing this medication should be vigilant about its rare toxicities.
